# Supplementary material for: Histopathological imaging features- versus molecular measurements-based cancer prognosis modeling
Source: Sci Rep. 2020 Sep 14;10:15030. doi: 10.1038/s41598-020-72201-5 (PMC7490375; doi:10.1038/s41598-020-72201-5)
Supplement: Supplementary file 2 — Supplementary information 2 [file 41598_2020_72201_MOESM2_ESM.docx]

**Histopathological imaging features- versus molecular measurements-based cancer prognosis modeling**

Sanguo Zhang^1^, Yu Fan^1,3^, Tingyan Zhong^2,3^ and Shuangge Ma^3,*^

^1^School of Mathematics Sciences, University of Chinese Academy of Sciences, Beijing 100049, China

^2^SJTU-Yale Joint Center for Biostatistics, Department of Bioinformatics and Biostatistics, School of Life Sciences and Biotechnology, Shanghai Jiao Tong University

^3^Department of Biostatistics, Yale School of Public Health, New Haven, CT 06520, USA

**Additional numerical analysis**

**(I) Analysis with additional marginal screening**

In some cancer studies with high dimensional variables (molecular, imaging, and others), marginal screening is conducted prior to analysis to reduce the data dimensionality to a more manageable level. In our analysis presented in the main text, as the dimensions are not as high, screening is not conducted. To be comprehensive, here we conduct a supervised marginal screening and select the top 50% imaging features and gene expressions with the highest significance in marginal Cox regression. The rest of the analysis is conducted in the same way as in the main text. The estimation results are as follows:

**Supplementary Table S1.** Analysis of LUAD data with marginal screening: identified imaging features and clinical characteristics associated with overall survival and their estimated coefficients.

| **Imaging feature** | **Coef** | **Clinical characteristic** | **Coef** |
| --- | --- | --- | --- |
| Count_identifytissueregion | 0.1588 | SEX | 0.0041 |
| AreaShape_Zernike_6_4 | 0.2742 | AGE | 0.0113 |
| Neighbors_AngleBetweenNeighbors_Adjacent | -0.2442 | tumor_size | 0.1781 |
| AreaShape_Zernike_8_6 | 0.0905 | A | -1.1802 |
| Threshold_WeightedVariance_identifyhemaprimarynuclei | 0.1193 | B | -0.3327 |
|  |  | C | NA |

*This is the counterpart of Table 2 in the main text.*

**Supplementary Table S2.** Analysis of LUSC data with marginal screening: identified imaging features and clinical characteristics associated with overall survival and their estimated coefficients.

| **Imaging feature** | **Coef** | **Clinical characteristic** | **Coef** |
| --- | --- | --- | --- |
| Granularity_9_ImageAfterMath | 0.1141 | SEX | 0.4489 |
| Granularity_12_ImageAfterMath | 0.2378 | AGE | 0.0235 |
| AreaShape_Center_X | -0.0787 | tumor_size | -0.1989 |
| Threshold_SumOfEntropies_identifytissueregion | 0.1656 | A | -0.7094 |
| AreaShape_EulerNumber | -0.1352 | B | -0.4947 |
| Location_Center_X.1 | -0.1182 | C | NA |

*This is the counterpart of Table 3 in the main text.*

**Supplementary Table S3.** Analysis of LUAD data with marginal screening: identified gene expressions and clinical characteristics associated with overall survival and their estimated coefficients.

| **Gene expression** | **Coef** | **Clinical characteristic** | **Coef** |
| --- | --- | --- | --- |
| RND3 | 0.2150 | SEX | 0.0244 |
| GLI2 | 0.2145 | AGE | 0.0170 |
| CCNB1 | 0.1462 | tumor_size | -0.0991 |
| RHOH | -0.1960 | A | -1.2279 |
| CTSL | 0.0684 | B | -0.3348 |
|  |  | C | NA |

*This is the counterpart of Table 4 in the main text.*

**Supplementary Table S4.** Analysis of LUSC data with marginal screening: identified gene expressions and clinical characteristics associated with overall survival and their estimated coefficients.

| **Gene expression** | **Coef** | **Clinical characteristic** | **Coef** |
| --- | --- | --- | --- |
| WDHD1 | -0.1518 | Sex | 0.3339 |
| MUC1 | 0.1250 | Age | 0.0241 |
| PRKCA | 0.2319 | Tumor_Size | -0.3872 |
|  |  | Stage_Level_A | -0.8592 |
|  |  | Stage_Level_B | -0.7606 |
|  |  | Stage_Level_C | NA |

*This is the counterpart of Table 5 in the main text.*

In the analysis that integrates the imaging and molecular data, for LUAD, the estimated regression coefficients are 0.8777 (imaging feature, p-value 6.98e-5) and 0.8741 (gene expression, p-value=8.68e-8). For LUSC, the estimated regression coefficients are 0.9678 (imaging feature, p-value=8.28e-7) and 0.7079 (gene expression, p-value=2.94e-3).

In the random-splitting based prediction evaluation, for LUAD, the median prediction C-index values are 0.6336 (imaging features), 0.6719 (gene expressions), and 0.6599 (combined). For LUSC, the median prediction C-index values are 0.5658 (imaging features), 0.5606 (gene expressions), and 0.5634 (combined).

Overall, it is found that this marginal screening leads to overlapping but also quite different estimation results. This is not surprising considering the significant different inputs. We note that the differences do not suggest a lack of stability of our analysis. Theoretically speaking, marginal screening is only valid under a set of stringent conditions. It has certain “contradictions” with the conducted joint analysis. The key finding is that for our particular data and analysis, marginal screening does not substantially change the observations on prediction performance.

**(II) Examination of the impact of tuning parameter selection**

The penalized estimations involve a tuning parameter, which is selected using cross validation – the default in *glmnet* and extensively adopted in the literature. Theoretical investigation on tuning parameter selection in high dimensional settings remains limited. It is even more challenging to decide whether a tuning parameter selection technique is appropriate with practical data. To get some insights into the impact of tuning parameter value, we identify the cross-validated optimal tunings, multiply by 0.95 and 1.05, and repeat analysis. This way, we can examine performance when the tuning parameters are not “too off” (otherwise, the analysis may not be meaningful).

In estimation, it is found that the identified imaging features and gene expressions have overlapping but also somewhat differ from those in Tables 2-5 of the main text. Details are omitted and available from the authors. (a) When the optimal tunings are multiplied by 0.95: in the integration analysis that combines the molecular and imaging data, for LUAD, we obtain regression coefficients 1.0513 (imaging feature, p-value=0.0007) and 1.0330 (gene expression, p-value=3.69e-11). For LUSC, we obtain regression coefficients 0.9577 (imaging feature, p-value=4.41e-05) and 0.6399 (gene expression, p-value=0.0003). In the random-splitting based prediction evaluation, for the LUAD data, the median prediction C-index values are 0.6297 (imaging features), 0.6949 (gene expressions), and 0.6945 (combined). For the LUSC data, the median prediction C-index values are 0.5721(imaging features), 0.5751 (gene expressions), and 0.5784 (combined). (b) When the optimal tunings are multiplied by 1.05: in the integration analysis that combines the molecular and imaging data, for LUAD, we obtain regression coefficients 0.8777 (imaging feature, p-value=6.98e-5) and 0.8741 (gene expression, p-value=8.68e-8). For LUSC, we obtain regression coefficients 0.9996 (imaging feature, p-value=1.66e-5) and 0.6334 (gene expression, p-value=0.0005). In the random-splitting based prediction evaluation, for the LUAD data, the median prediction C-index values are 0.6106 (imaging features), 0.6879 (gene expressions), and 0.6752 (combined). For the LUSC data, the median prediction C-index values are 0.5516 (imaging features), 0.5664 (gene expressions), and 0.5625 (combined).

Overall, it is observed that tuning parameter values have an impact on estimation and selection, as expected. The impact on prediction performance is limited. As tuning parameter selection remains a not-completely-solved problem in the literature, we choose not to explore further.
